# System metabolic engineering of *Escherichia coli* W for the production of 2-ketoisovalerate using unconventional feedstock

**DOI:** 10.3389/fbioe.2023.1176445

**Published:** 2023-04-20

**Authors:** Darwin Carranza-Saavedra, Jesús Torres-Bacete, Blas Blázquez, Claudia Patricia Sánchez Henao, José Edgar Zapata Montoya, Juan Nogales

**Affiliations:** ^1^ Faculty of Pharmaceutical and Food Sciences, Nutrition and Food Technology Group, University of Antioquia, Medellín, Colombia; ^2^ Department of Systems Biology, National Centre for Biotechnology (CSIC), Systems Biotechnology Group, Madrid, Spain; ^3^ Interdisciplinary Platform for Sustainable Plastics Towards a Circular Economy‐Spanish National Research Council (SusPlast‐CSIC), Madrid, Spain

**Keywords:** dairy by-products, L-valine, bioeconomy, feedback inhibition, non-conventional microbial factories, systems biotechnology

## Abstract

Replacing traditional substrates in industrial bioprocesses to advance the sustainable production of chemicals is an urgent need in the context of the circular economy. However, since the limited degradability of non-conventional carbon sources often returns lower yields, effective exploitation of such substrates requires a multi-layer optimization which includes not only the provision of a suitable feedstock but the use of highly robust and metabolically versatile microbial biocatalysts. We tackled this challenge by means of systems metabolic engineering and validated *Escherichia coli* W as a promising cell factory for the production of the key building block chemical 2-ketoisovalerate (2-KIV) using whey as carbon source, a widely available and low-cost agro-industrial waste. First, we assessed the growth performance of *Escherichia coli* W on mono and disaccharides and demonstrated that using whey as carbon source enhances it significantly. Second, we searched the available literature and used metabolic modeling approaches to scrutinize the metabolic space of *E. coli* and explore its potential for overproduction of 2-KIV identifying as basic strategies the block of pyruvate depletion and the modulation of NAD/NADP ratio. We then used our model predictions to construct a suitable microbial chassis capable of overproducing 2-KIV with minimal genetic perturbations, i.e., deleting the pyruvate dehydrogenase and malate dehydrogenase. Finally, we used modular cloning to construct a synthetic 2-KIV pathway that was not sensitive to negative feedback, which effectively resulted in a rerouting of pyruvate towards 2-KIV. The resulting strain shows titers of up to 3.22 ± 0.07 g/L of 2-KIV and 1.40 ± 0.04 g/L of L-valine in 24 h using whey in batch cultures. Additionally, we obtained yields of up to 0.81 g 2-KIV/g substrate. The optimal microbial chassis we present here has minimal genetic modifications and is free of nutritional autotrophies to deliver high 2-KIV production rates using whey as a non-conventional substrate.

## 1 Introduction

Success in the implementation of a fully circular economy demands sustainable alternatives to substrates traditionally used in fermentative processes to avoid competition with human and animal food, e.g., glucose. Fulfilling this simple and yet very ambitious goal is the challenge driving interest in the use of agricultural and agro-industrial waste as feedstock for large-scale fermentation processes. However, non-conventional carbon sources often deliver lower yields due to their limited degradability and the presence of toxic byproducts (Ren et al., 2011; Lopes et al., 2019). Overcoming these challenges therefore requires multi-layer optimization to deliver suitable alternative feedstocks and non-conventional, highly robust and metabolically versatile microbial biocatalysts.

In recent years, much research has been conducted into the use of whey as a fermentation medium to produce lipids, organic acids and alcohols (Cao et al., 2020; Mano et al., 2020; Novak et al., 2020). The physicochemical composition of whey is behind this growing interest, as it typically contains around 88% lactose, 4% protein, 1.4% (w/w) lipids and trace minerals in powder product (Carranza-Saavedra, Sánchez Henao and Zapata Montoya, 2021). While it is usually more expensive to grow bacteria on pure lactose than on glucose, using whey as a source of lactose significantly reduces the cost (Silva et al., 2015; Amado et al., 2016), thus making whey a promising non-conventional feedstock for microbial biotechnology.

Searching for a new non-conventional bacterial chassis able to deal with such a novel feedstock while providing additional metabolic and/or genetic advantages is also a challenge. Strains free from catabolic repression phenomena, among other features, would be the ideal candidates (Calero and Nikel, 2019). The *Escherichia coli* W strain has been used previously to produce natural metabolites and it brings potential improvements over *E. coli* K-12 strains: 1) it is able to use a broader range of carbon sources, at high concentration, 2) it is the only safe *E. coli* strain that uses sucrose as a carbon source (Archer et al., 2011; Felpeto-Santero et al., 2015; Erian et al., 2018), 3) it releases smaller amounts of acetate during fermentation while generating more biomass in batch culture (Archer et al., 2011), and 4) it tolerates increased stress conditions, such as high ethanol concentrations, acidic pH, high temperature and osmotic stress (Shiloach and Bauer, 1975; Gleiser and Bauer, 1981; Alterthum and Ingram, 1989; Nagata, 2001).

On the other hand, despite the long history of success using traditional metabolic engineering strategies, successful integration of modern biotechnology into the circular economy requires the development of new holistic approaches and tackling the complexity of living organisms from a systems-level perspective. The combination of computational methods based on metabolic models and cutting-edge synthetic biology has so far delivered a high degree of success in metabolic engineering endeavors. Indeed, it has paved the way for systematic exploration of the metabolic solution spaces required to produce target metabolites (Wu et al., 2016; Gudmundsson and Nogales, 2021). Design-Build-Test-Learn (DBTL) iterative cycles stand up as popular examples of such multidisciplinary approaches to the production of a plethora of chemical compounds (Gurdo et al., 2022). DBTL cycles are iterative designs combining the advances in systems and synthetic biology to deliver rational genetic modifications and high-throughput phenotyping of strains. DBTL-driven research leads to the establishment of sound conclusions from experimental results, knowledge building and the generation of new hypotheses. Hence, it contributes to unlocking novel biotechnological processes supporting the circular economy.

**FIGURE 1:**
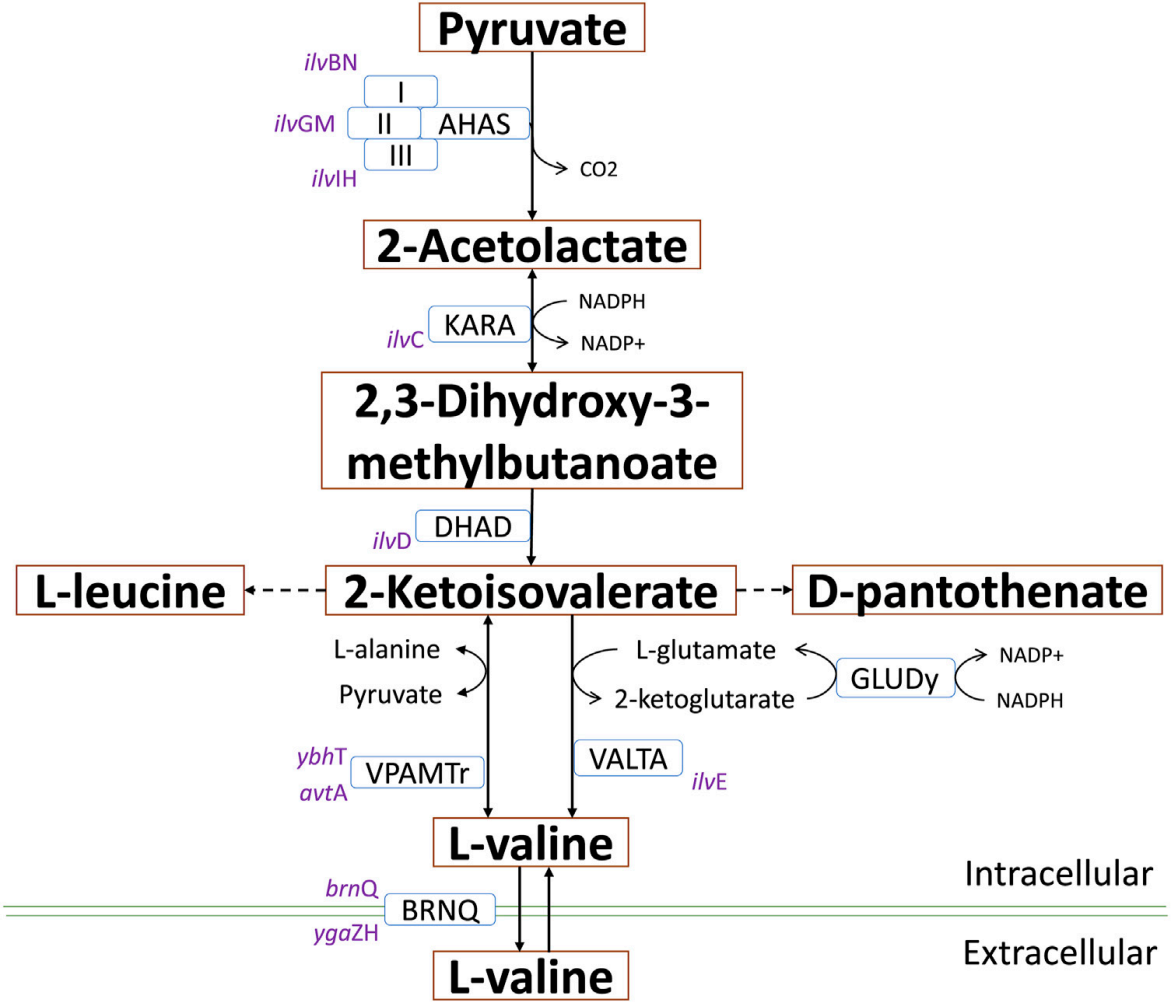
L-valine biosynthesis in *E. coli* via 2-KIV. Acetohydroxyacid synthase (AHAS), ketol-acid reductoisomerase (AHAIR), dihydroxyacid dehydratase (DHAD), valine transaminase (VALTA), valine-pyruvate aminotransferase (VPAMTr), branched-chain amino acid transport system 2 carrier protein (BRNQ). Gene names are shown in purple.

In this context, an interesting idea is to apply multidisciplinary approaches to optimize biotechnological platforms towards the cost-effective production of key building blocks, which in turn are the precursors of a variety of value-added compounds. 2-ketoisovalerate (2-KIV) is one of these key building blocks and it has been the focus of significant attention (Gu et al., 2017). 2-KIV is an important precursor in the biosynthesis of branched chain amino acids (BCAAs) such as L-valine, cofactors such as pantothenate, coenzyme A and other biologically active compounds such as glucosinolates (Felnagle et al., 2012; Lee et al., 2019; Zhao et al., 2022). In microorganisms and plants, synthesis of 2-KIV requires two pyruvate molecules via three consecutive reactions included in the BCAAs pathway which are catalyzed by acetolactate synthase (AHAS), ketol-acid reductoisomerase (KARA) and dihydroxy-acid dehydratase (DHAD) (Figure 1). Production of 2-KIV has been addressed using *Klebsiella pneumoniae* (Gu et al., 2017), *Corynebacterium glutamicum* (Buchholz et al., 2013), *Pseudomonas putida* (Batianis et al., 2022) and, more recently, *E. coli* (Zhou et al., 2022). 2-KIV production with *E. coli* has also been addressed extensively in the context of L-valine and isobutanol production (Blombach et al., 2007; 2011; Park et al., 2007; Atsumi et al., 2008; Holátko et al., 2009; Krause et al., 2010; Park et al., 2011a; Bartek et al., 2011; Park et al., 2011b; Li et al., 2011; Hou et al., 2012; Lee et al., 2012; Buchholz et al., 2013; Hasegawa et al., 2013; Chen et al., 2015; Gu et al., 2017; Liang et al., 2018; Schwentner et al., 2018; Westbrook et al., 2018; Noda et al., 2019; Hao et al., 2020; Jung et al., 2020). However, such studies used conventional feedstocks and highly engineered strains harboring multiple autotrophies, thus requiring complex and expensive production media to support the bioprocess.

In this study, we used systems and synthetic biology to produce 2-KIV in a microbial factory fueled by whey and driven by the non-conventional and promising *E. coli* W strain. The strain’s design featured minimal genetic intervention in order to preserve its growth performance in minimal medium without the addition of expensive nutritional supplements. 24 h batch culture assays returned a 3.22 ± 0.07 g/L titer for 2-KIV and 1.40 ± 0.04 g/L for L-valine. We also obtained yields (Yp/s) of up to 0.8 g/g for 2-KIV.

## 2 Materials and methods

### 2.1 Strains, plasmids, primers and DNA parts

Bacterial strains and plasmids used in this work are listed in Table 1. Additional biological material including primers, DNA parts (expression systems, promoters, RBSs, CDSs, terminators and host plasmids can be found in Supplementary Tables S1–S3).

**TABLE 1:** Strain, plasmids and DNA used in this study.

Strain	Genotype	Source
*E. coli* DH5α	fhuA2 Δ(argF-lacZ)U169 phoA glnV44 Φ80 Δ(lacZ)M15 gyrA96 recA1 relA1 endA1 thi-1 hsdR17	New England BioLabs (NEB)
*E. coli* W	Wild Type (ATCC 9637) (WT)	www.atcc.org
WT Δmdh-aceF	*E. coli* W, Δmdh-ΔaceF	This study
*E. coli* W1288	*E. coli* W, Δmdh-ΔaceF, pSEVA681	This study
*E. coli* W1294	*E. coli* W, pSEVA681-kiv	This study
*E. coli* W1262	*E. coli* W, Δmdh-ΔaceF, pSEVA681-kiv	This study
Plasmid	Description	Source
pSLTS	ori SC101(Ts) Apr; ParaB for λ-Red; PtetR for I-SceI Gama, beta, exo	Kim et al. (2014)
pSEVA182	Amp^r^, pUC ori, cargo: lacZα-pUC19	Martínez-García et al. (2022)
pSBG422	I-SceI site and a kanamycin resistance gene with SacI/BamHI sites and SacI/BamHI pUC ori Km and Ap	
pSEVA681	Gm^r^, pUC ori (cloning vector of high copy number), cargo: MCS-default	
pKIV	Gm^r^, pUC ori, CDS: XylS/Pm- *als*S-*ilv*D-*ilv*C, RBS-ST, BBa_B1006 terminator and linkers	This study

### 2.2 Reagents and culture media

Spray-dried, food grade whey powder containing 88.08% ± 1.93% lactose, 4.16% ± 0.12% protein and 1.46% ± 0.25% fat (w/w) was purchased from Cimpa S.A.S. (Bogotá, Colombia). Other reactive agents used were analytical grade. *E. coli* strains grown in Luria-Bertani (LB) medium and Minimal medium M9 (pH 7.2) (Landor et al., 2023) with carbon source concentrations set at 2 g/L. Under production conditions, M9 was supplemented with 1 g/L of yeast extract and 10 g/L of carbon source. We prepared a stock solution for whey-based fermentation media mixing 10 g of whey powder in 100 mL of distilled water. All culture media and work solutions were sterilized at 121°C for 15 min and their initial pH adjusted to 7.0 before use.

### 2.3 DNA manipulation

We used *E. coli* DH5α as the cloning host for plasmid and cassette construction and *E. coli* W as the starting strain for genomic manipulations. We purchased DNA polymerases and other DNA-modifying enzymes from New England Biolabs (Ipswich, United States) and oligonucleotides from Sigma-Aldrich (Madrid, Spain). We sourced DNA purification kits from Nyztech (Lisbon, Portugal). We ran PCRs on a Mastercycler Nexus Gradient thermal cycler (Eppendorf, Germany) and analyzed PCR products with 0.7 or 1.5% agarose gel-electrophoresis using a 1X Tris-acetate buffer. Antibiotics, i.e., ampicillin (100 μg/mL), kanamycin (50 μg/mL) and gentamicin (10 μg/mL) were added to culture media and agar plates according to assay requirements.

*ace*F and *mdh* genes were deleted by using scarless genome editing as described by Kim et al. (2014) with slight modifications. 300 DNA-base pair plasmids corresponding to homologous regions of the *E. coli* W genome were designed and pSEVA182_aceF_Arm1y2 and pSEVA182_mdh_Arm1y2 cassettes delivered by means of digestion with type II restriction enzymes and ligation with T4 ligases (Supplementary Figures S1, S2; cassette sequences in Supplementary Table S3). Further details are available in Supplementary MD1. Supplementary Figure S3 shows PCR and gel electrophoresis verification of mutations.

We assembled a synthetic expression system using a modular cloning technique based on Golden Standard (Blázquez et al., 2022; Martínez-García et al., 2022). Simultaneous design of overexpression systems supported combinatorial, multi-part assembly of standardized genetic elements to deliver genetic circuits. We designed the pKIV plasmid using the 3-methyl-benzoate (3 MB)-activated XylS/Pm expression system (Gawin et al., 2017). Detailed descriptions of synthetic pathway construction are available in Supplementary MD2.

### 2.4 Production in shake flasks

We inoculated strains in 25 mL LB medium (seed culture) in 250 mL shake flasks and incubated overnight at 37°C in a MaxQ 4000 incubator shaker (Thermo Scientific, United States) at 250 rpm. Seed cultures were centrifuged (5,200 g), washed with NaCl (0.85% w/v) and inoculated in 25 mL of growth medium (preinoculum) at 37°C and 250 rpm for 24 h. Preinocula were washed with NaCl and transferred to a 250 mL shake flask containing 20 mL of fermentation medium for 24 h-cultivation at 37°C, 250 rpm shaking and initial optical density (OD_600nm_) set to 1.0. 0.5 mM 3 MB inductor was added to the fermentation medium. We analyzed the cellular toxicity due 2-KIV and did not observe negative effect on growth at concentration ranging from 0 to 5 mM (data not shown).

### 2.5 System-level analysis of sugar metabolism

To assess growth kinetics, cells were cultivated at 37°C for 13 h in a growth medium containing a mixture of glucose, lactose, galactose, sucrose, fructose and maltose at a concentration of 2 g/L as carbon source. Initial OD_600nm_ was 0.1 and shaking was set to 250 rpm. Besides, each of the sugars was mixed with glucose at a 50:50 glucose:target sugar ratio (concentration in the mix was 2 g/L). Whenever we used sucrose, *E. coli* W was grown twice in M9 supplemented with sucrose before running growth kinetics assays.

### 2.6 Analytical techniques

We measured OD_600nm_ with a Genesys 10S UV-Vis spectrophotometer (Thermo Scientific, U.S.A) to quantify biomass. OD_600nm_ readings were correlated with dry cell weight per liter (g DCW/L) using the equation: g DCW/L = OD_600nm_*0.452 (Erian et al., 2018).

Sugar concentrations (glucose and lactose) were quantified by spectrophotometric techniques using the DNS method (Carranza-Saavedra et al., 2021). We measured sample absorbance at 540 nm wavelength in a Genesys 10S UV-Vis spectrophotometer (Thermo Scientific, U.S.A). We then used glucose and lactose concentrations ranging between 0.5 and 8 g/L to plot calibration curves for both sugars.

We used HPLC to measure organic acid and L-valine concentrations. All samples were carefully filtered using a 0.2 µm cellulose nitrate syringe filter before being injected into the HPLC. Analyses were carried out using a Thermo Ultimate 3000 HPLC system (Thermo Fisher Scientific, United States) equipped with a quaternary pump, an automatic injector, a column thermostat set and a diode arrangement detector with UV detection. Following the method described by Kerem et al. (Kerem et al., 2004), a Zorbax Bonus-RP Column 4.6 × 250 mm (Agilent, U.S.A), particle size 5 μm, was used to quantify 2-KIV. We used two solvents as eluents for gradient elution: solvent A was trifluoroacetic acid (0.2% in water, pH 1.96) and solvent B was acetonitrile. Injection volume was 20 μL and 10 min equilibration intervals (solvent A) were required between injections. Elution started at 0.8 mL/min with an isocratic step of solvent A (15 min), then a solvent B linear gradient (0%–50%) for the next 5 min and, finally, 5 min with 80% solvent B. Column temperature was set to 30°C and detection wavelength to 210 nm. We used organic acid concentrations from 3 to 30 mM to plot a calibration curve (external standard).

Amino acids (L-valine) were separated in a Zorbax Eclipse AAA-C18 column 4.6 × 75 mm (Agilent, United States), particle size 3.5 µm, with a Zorbax Eclipse AAA pre-column 4.6 × 12.5 mm (Agilent, United States), particle size 5 µm and quantification following the procedure described by Cigić, et al. (Cigić et al., 2008). The column thermostat was set to 40°C and detection wavelength to 338 nm to quantify primary amino acids. We pre-derivatized *in situ* with ortho-phthalaldehyde and 3-marcaptopropionic acid (OPA/3-MPA). An amino acids standard curve (0.5, 1.0, 1.5 and 2.0 μmol/mL) was used for quantification (external standard).

### 2.7 Kinetic parameters

We calculated maximum specific growth rate (µ_max_) by linear regression of the natural logarithm of g DCW/L over time (Eq. 1) during the exponential phase. Substrate yields in biomass (Eq. 2), substrate in product (Eq. 3) and biomass in product (Eq. 4) were calculated using the relationship between the concentration of resulting dry biomass, the concentration of consumed glucose and total product concentrations, respectively.

$${µ}_{\max}=\frac{dX}{Xdt}$$ (1)

$$Y_{X/S}=\frac{dX}{dS}$$ (2)

$$Y_{P/S}=\frac{dP}{dS}$$ (3)

$$Y_{P/X}=\frac{dP}{dX}$$ (4)

### 2.8 Constraints-based analysis

The *E. coli* W iCA_1273 metabolic model was download from the BioModels Database (MODEL1507180010) (https://www.ebi.ac.uk/biomodels/). iCA_1273 is a manually curated and validated metabolic model (Archer et al., 2011) including 2,477 reactions and 1,111 metabolites encoded by 1,273 genes. Our analyses required adding the ability to secrete 2-KIV to the model, which resulted in a new model featuring 2,480 reactions. We used flux balance analysis (FBA) (Heirendt et al., 2019) to interrogate the model and characterized flux distribution through the network of reactions with Eq. 5.

S*v=0 (5)

Where **S** is the dimension of the stoichiometric matrix given by **m** (number of metabolites in the reaction network) and **n** (number of reactions). **v** indicates the flow rate through each of the reactions.

*In silico* analysis was performed with MATLAB R2018a, the COBRA Toolbox v.3.0 and Gurobi solver (version 9.5.1). We used Monte Carlo sampling to register frequency and flux variability along pathways showing potential increases in pyruvate, 2-KIV and L-valine production. Samples were collected from 35,000 points over 4 h (previous test) (Heirendt et al., 2019). Reactions assessed using Monte Carlo sampling are listed in Supplementary Table S4.

### 2.9 Statistical analysis

All shake flask experiments were performed in duplicate and data obtained were expressed as mean ± standard error. Kinetic parameters obtained from experiments were analyzed against each other using Duncan’s multiple comparison (Duncan, 1955) with a confidence level of 95%. Our aim was to establish potential significant differences in performance and relate them to the carbon sources used in growth media.

## 3 Results and discussion

### 3.1 Systems-level analysis of complex sugar metabolism in *E. coli* W

**FIGURE 2:**
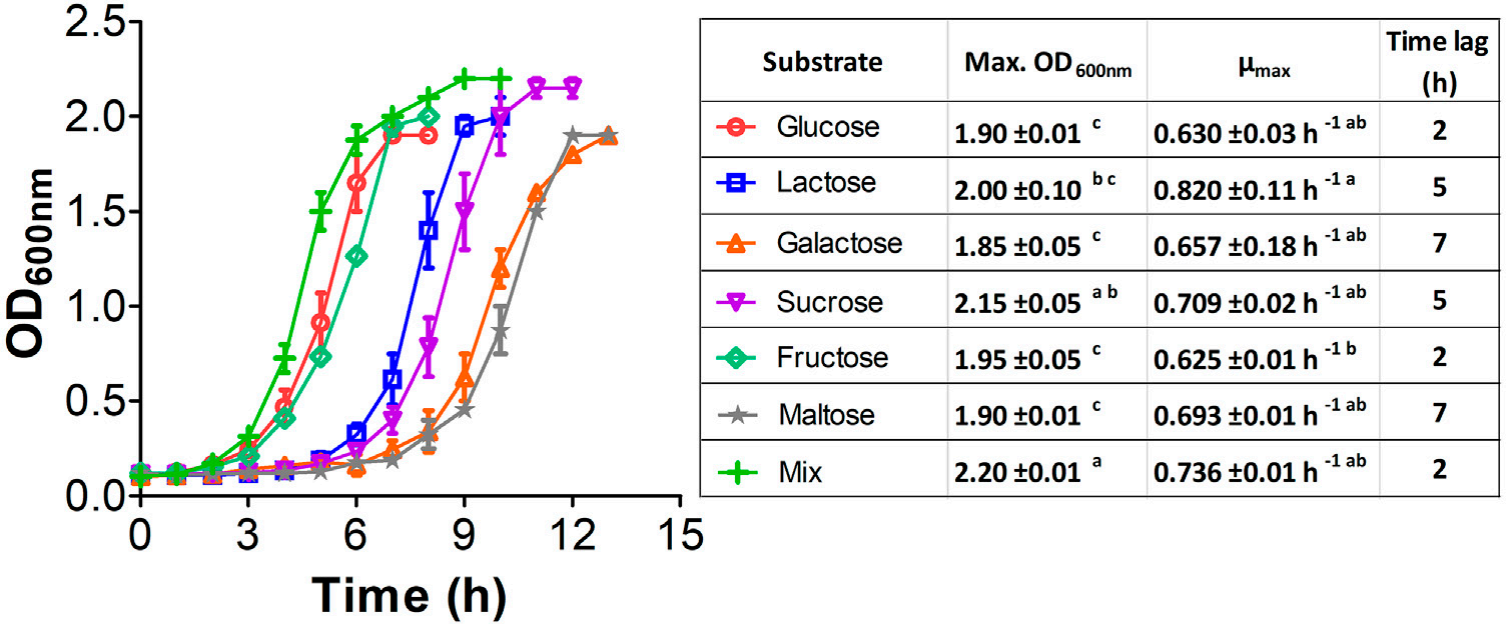
Growth kinetic of *E. coli* W using a variety of mono and disaccharides as sole carbon and energy source. 2 g/L of each single carbon source were used while for the mixture, a total of 0.33 g/L of each compound were used. Vertical bars, ± represent standard deviation and lines are a guide for the eye. Similar lowercase letters per column indicate no statistical difference between treatments (*p* < 0.05).

It is well-known that *E. coli* W has a complex sugar metabolism and is able to use several types of carbohydrate as sole carbon and energy sources (Wang et al., 2019). To assess the metabolic performance of *E. coli* W growing on such compounds, we monitored the growth kinetics of cultures feeding on a variety of monosaccharides (glucose, fructose and galactose), disaccharides (lactose, sucrose and maltose) and a mixture of them all. Glucose and fructose were rapidly assimilated (Figure 2), so they turned out to support the shortest lag phase. They were followed by lactose and sucrose, while galactose and maltose displayed the longest lag phases. Despite their longer latency periods, disaccharides provided the highest growth rates (lactose > sucrose > maltose) and final OD_600_ (sucrose > lactose > maltose). Interestingly, we observed the shortest latency period, highest growth rate and greatest final biomass with the sugar mix, which strongly suggests a synergistic and complementary metabolism supporting fast nutrient consumption and growth. The absence of diauxic growth curves strongly argues in favor of a limited effect of catabolic repression in the metabolism of mixture of sugars in this strain. These results show not just that *E. coli* W is able to use a variety of carbohydrates as single carbon and energy sources efficiently, but more critically that the availability of a carbohydrate mix has a positive impact on its growth performance. In addition, negligible catabolic repression (CCR) is an advantage over other conventional *E. coli* strains like *E. coli* K12, which suffered from the negative impacts of CCR in experiments using glucose and non-PTS sugars as carbon source (Luo et al., 2014).

**FIGURE 3:**
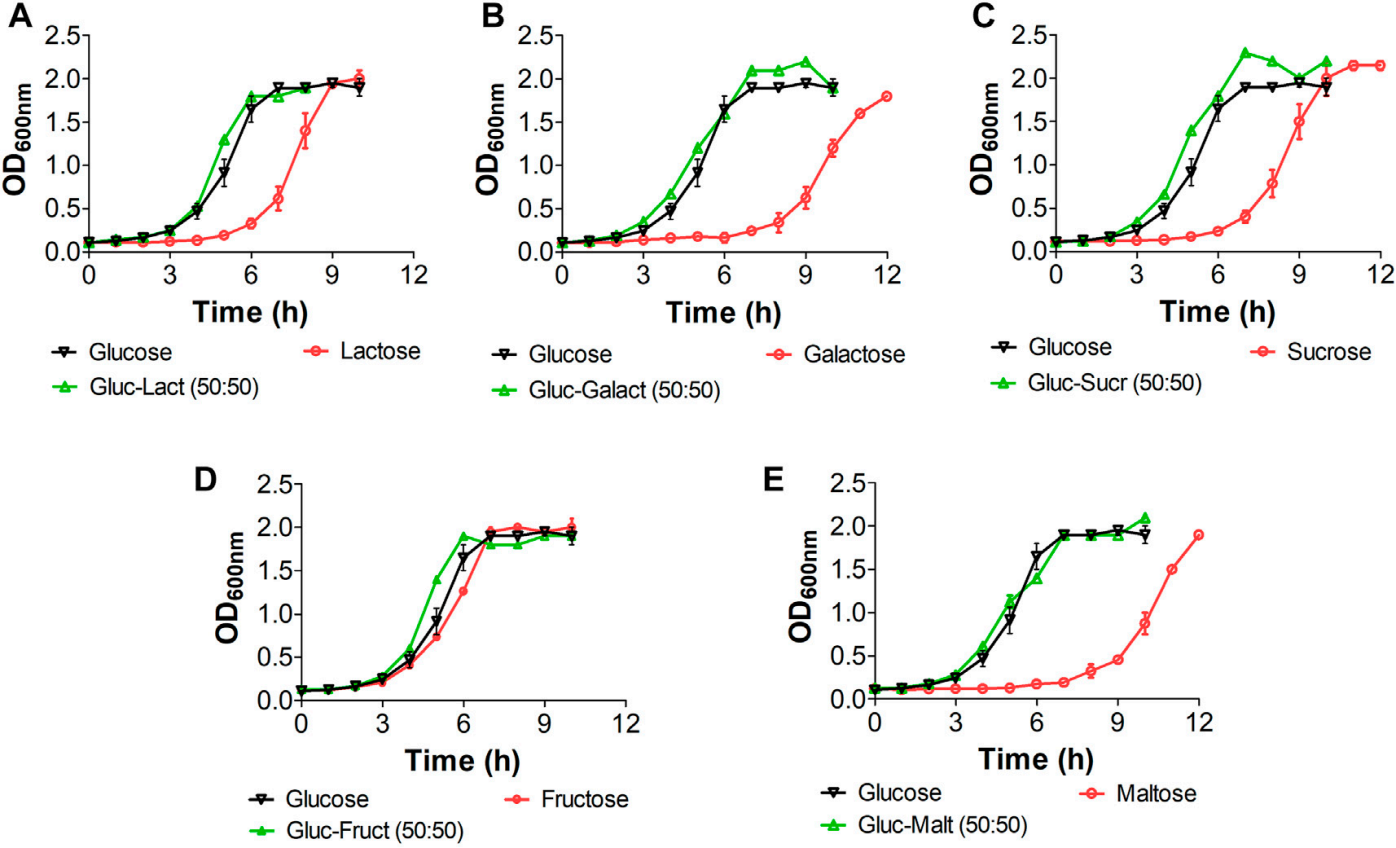
Growth kinetic of *E. coli* W in mix sugars with glucose for studying diauxic growth. All mix and pure carbon source were used at of 2 g/L. Vertical bars represent standard deviation and lines are a guide for the eye. **(A)**: Kinetic with lactose, **(B)**: kinetic with galactose, **(C)**: kinetic with sucrose, **(D)**: kinetic with fructose, **(E)**: kinetic with maltose.

We implemented a series of growth experiments using glucose at different ratios as an additional carbon source to further assess the role of CCR in *E. coli* W and verify whether its growth performance improves when using carbohydrate mixes instead of single carbon sources (Figure 3). Although there is no evidence of a marked diauxic behavior in our kinetics assessment, µ_max_ analysis for each of the different substrates (Supplementary Table S5) shows a slight affectation when using glucose with galactose and maltose (Figures 3B, E). However, there are no significant differences (*p* < 0.05) in µ_max_ between other glucose-containing mixes. Significant differences between glucose-lactose mixes and other mixes, particularly when they contained glucose, show that the former favor growth. Overall, our results suggest that sugar-mix-powered *E. coli* W microbial cell factories are very promising, not just because they are virtually free of CCR but because their growth performance is improved when multiple sugars are simultaneously available.

### 3.2 Legacy and model-based design of a set of 2-KIV overproducer *E. coli* W strains

*E. coli* W’s efficient carbohydrate metabolism ensures a large carbon flux around pyruvate, a key metabolic hub in sugar catabolism and the main precursor of 2-KIV. However, cost-effective production of pyruvate-derived metabolites such as 2-KIV requires additional flux rerouting (Gu et al., 2017; Noda et al., 2019). Nowadays, redirection of carbon flux towards pyruvate has been unlocked in a large variety of microorganisms (Buchholz et al., 2013; Gu et al., 2017; Novak et al., 2020), although most studies have focused on disabling pyruvate consumption along competing pathways (Noda et al., 2019; Novak et al., 2020). For instance, detailed analysis of studies dealing with overproduction of 2-KIV from glucose (Supplementary Figure S4; Supplementary Table S6) revealed three main knockout strategies. The first is the preferred choice and it avoids consumption of pyruvate as a precursor of: 1) acetyl-CoA via the pyruvate dehydrogenase complex (PDH, *ace*EF) or pyruvate formate lyase (PFL, *pfl*ABCD), 2) acetate via pyruvate oxidase (PO, *pox*B), 3) lactate via lactate dehydrogenase (LDH, *ldh*A), 4) L-alanine via glutamate-pyruvate aminotransferase (GPA, *yfd*ZQ) and 5) L-valine via branched-chain amino acid aminotransferases (*ilv*E) (Wang et al., 2018). Deletion of PDH seems to be of critical importance and is thus the most widely deleted competing pathway to increase levels of pyruvate in the cell (Supplementary Figure S4) (Blombach et al., 2007; 2011; Park et al., 2007; Park et al., 2011a; Bartek et al., 2011; Buchholz et al., 2013; Chen et al., 2015; Nitschel et al., 2020). Alternatively, deleting anaplerotic reactions such as phosphoenolpyruvate carboxylase (PPC, *ppc*) reduces gluconeogenesis from pyruvate and therefore delivers higher levels of 2-KIV (Buchholz et al., 2013; Hasegawa et al., 2013; Schwentner et al., 2018). Beyond blocking pyruvate-consuming pathways, the second strategy is aimed at unbalancing the NAD/NADP ratio via deletion of malate dehydrogenase (MDH, *mdh*) (Park et al., 2007; Park et al., 2011b) or redirecting the flux towards PPP via deletion of phosphate glucose isomerase (PGI, *pgi*). This has been often used to increase the carbon flux through the branched chain amino acid (BCAA) pathway (Park et al., 2007; Park et al., 2011a; Bartek et al., 2011; Noda et al., 2019). Finally, deleting metabolite-specific competing reactions in order to streamline the optimized pathway is also a recurrent strategy resulting in improved titers (Supplementary Figure S4). For instance, efficient production of L-valine required blocking the biosynthesis of alternative 2-KIV derivatives, such as L-leucine and/or pantothenate (Radmacher et al., 2002; Park et al., 2007; Holátko et al., 2009; Park et al., 2011b; Westbrook et al., 2018). However, these deletions resulted in auxotroph strains requiring complex culture media to grow, thus increasing the operational cost of the bioprocess.

**FIGURE 4:**
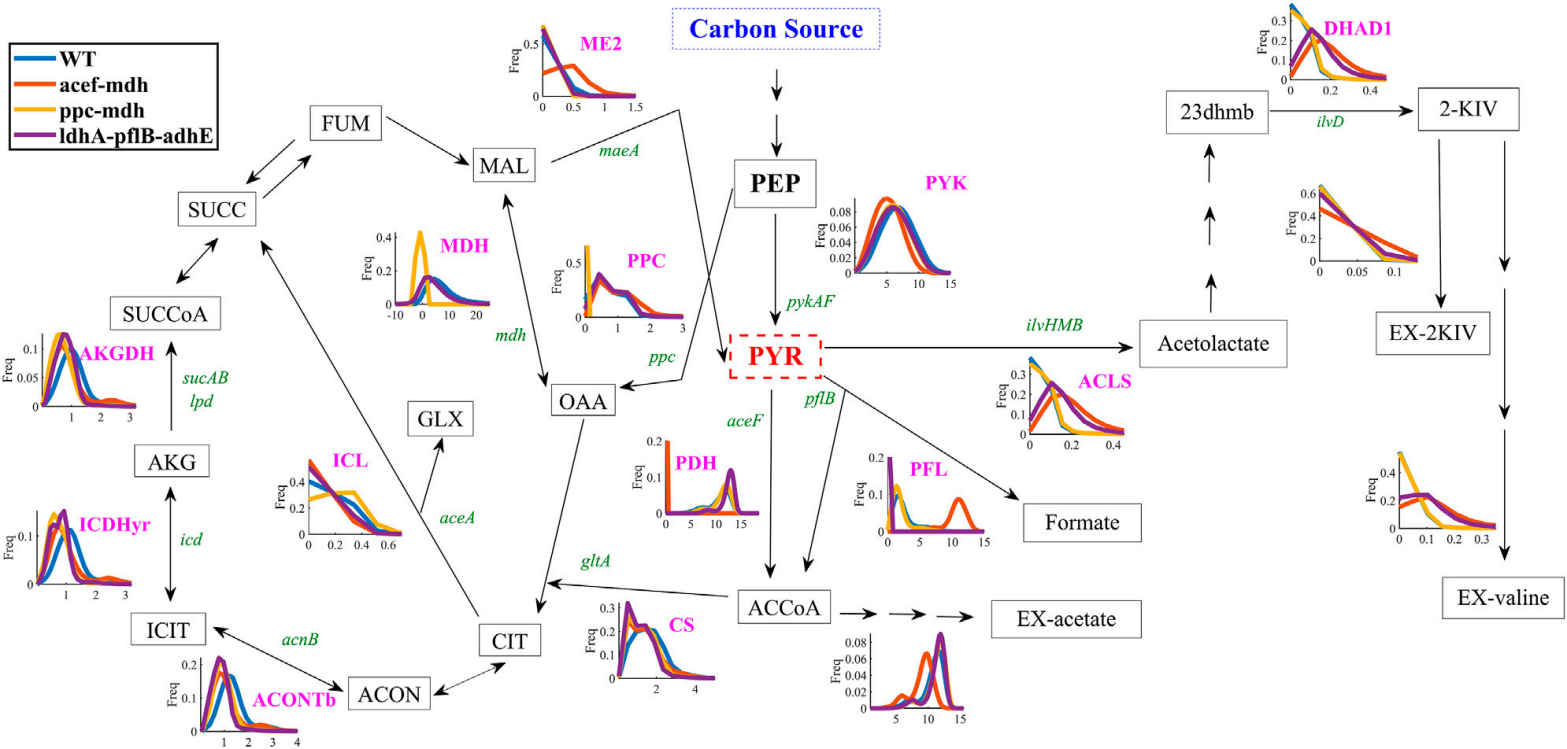
Flux frequency and variability through central metabolism of *E. coli* W as predicted by Monte Carlo sampling. Pyruvate kinase reaction (PYK), acetolactate synthase reaction (ACLS), dihydroxy-acid dehydratase reaction (DHAD1), extracellular transport of 2-KIV (EX-2KIV), extracellular transport of L-valine (EX-valine), malate dehydrogenase ubiquinone-8 reaction (ME1-2), pyruvate dehydrogenase reaction (PDH), phosphoenolpyruvate carboxylase reaction (PPC), malate dehydrogenase reaction (MDH), extracellular transport of acetate (EX-acetate), extracellular transport of formate (EX-formate), isocitrate lyase reaction (ICL), isocitrate dehydrogenase—NADP reaction (ICDHyr), oxogluterato deshidrogenasa reaction (AKGDH), pyruvate formate lyase (PFL), aconitase (ACONTb), citrate synthase (CS), fumarate (FUM), malate (MAL), oxaloacetate (OAA), succinate (SUCC), succinyl-CoA (SUCCoA), 2-oxoglutarate (AKG), isocitrate (ICIT), cis-aconitate (ACON), citrate (CIT), glyoxylate (GLX), phosphoenolpyruvate (PEP), pyruvate (PYR), Acetyl-CoA (ACCoA), 2,3-Dihydroxy-3-methylbutanoate (23 dhmb). Gene names are shown in green.

It seems reasonable to minimize the number of knockouts while maintaining high production in minimal medium to deliver a cost-effective 2-KIV production bioprocess. Therefore, we assessed 2-KIV production *in silico* to measure the performance of a set of knockout scenarios harboring minimal deletions. These included the removal of pyruvate-consuming pathways and generation of cofactor imbalances. Specifically, we used Monte Carlo Sampling to explore the metabolic space of three *in silico* mutant strains, i.e., *ace*F-*mdh*, *ppc*-*mdh* and *ldh*A-*pfl*B-*adh*E (Figure 4). Selection of this set of reactions was based on the outcomes of previous studies (Supplementary Table S6; Supplementary Figure S4) using caution to avoid auxotrophies in the final chassis.

Quantitative analysis of flux frequency shows that, of all tested mutants, the double *ace*F*-mdh in silico* strain exhibited the highest flux through the ACLS, DHAD and Ex-2KIV reactions (Figure 4). These results strongly suggest an increased flux through the BCAA pathway, which is in agreement with results from previous studies dealing with L-valine production (Park et al., 2007). Similarly, the *ace*F-*mdh* double mutant has a higher frequency and metabolic flux through the ME2 reaction. This should significantly contribute not only to providing additional levels of NADPH to the KARA reaction (Figure 1), but also to increasing pyruvate levels (Noronha et al., 2000), thus resulting in higher levels of 2-KIV. Finally, the double *ace*F*-mdh* mutant also exhibited a significantly lower acetate secretion compared to the other mutants, which greatly enhances this strain’s potential. Overall, we found that the double *ace*F*-mdh* was the most promising.

### 3.3 Carbon flux rerouting towards pyruvate promotes L-valine production in *E. coli* W

**FIGURE 5:**
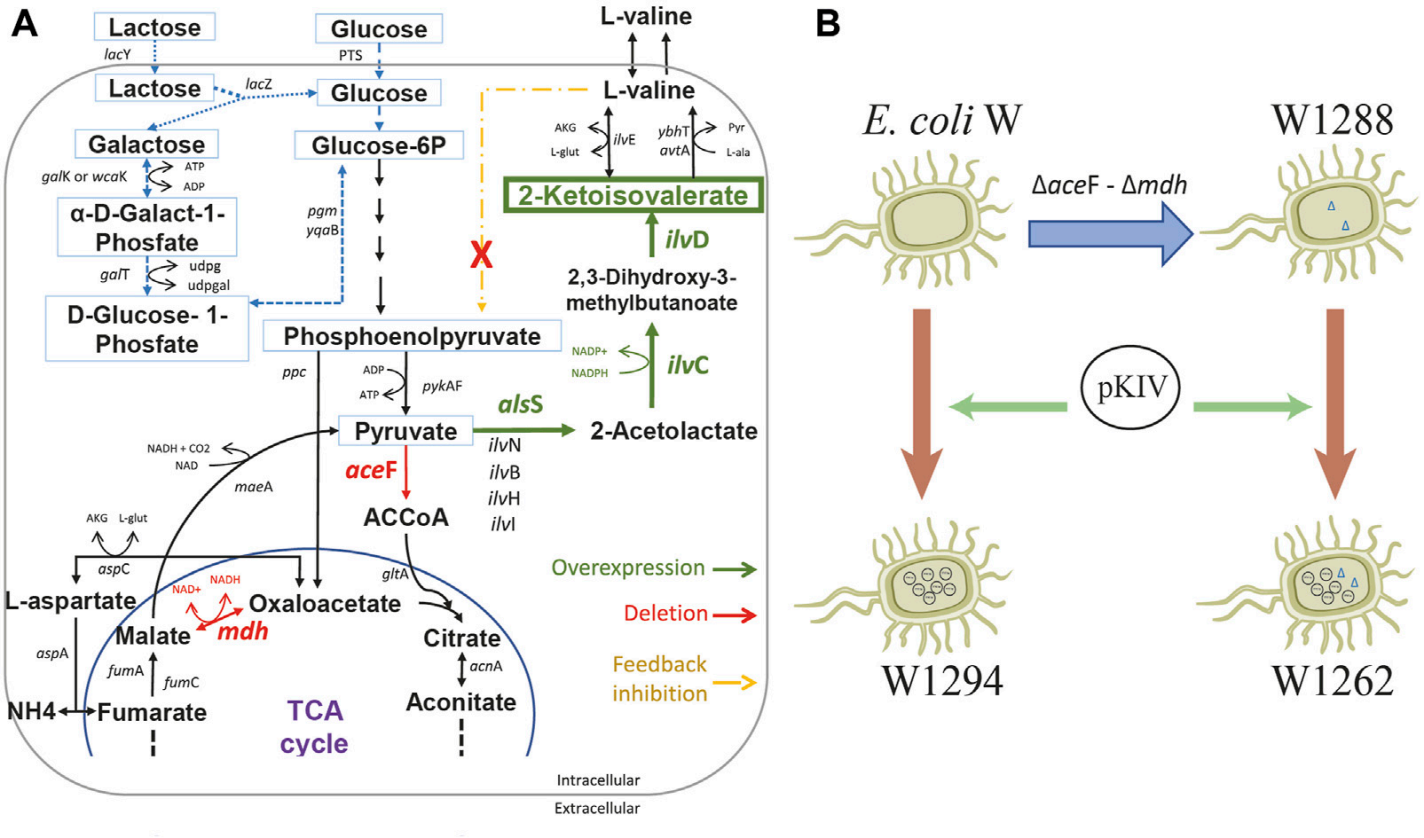
Schematic diagram of *E. coli* W engineering for 2-ketoisovalerate (2-KIV) overproduction. **(A)**: Main genetic interventions addressed to construct a 2-KIV overproducer *E. coli* W strain, **(B)**: Engineering *E. coli* W strains. Phosphotransferase system (PTS), Glucose-6-phosphate (Glucose-6P), acetylcoenzyme A (ACCoA), Tricarboxylic Acid Cycle (TCA cycle). Gene names are shown in italic.

We constructed a 2-KIV biotechnological platform comprising three major stages: 1) deletion of the *ace*F and *mdh* genes to re-route the carbon flux*,* 2) overexpression of the 2-KIV biosynthesis pathway and 3) removal of L-valine negative feedback (Figure 5).

**FIGURE 6:**
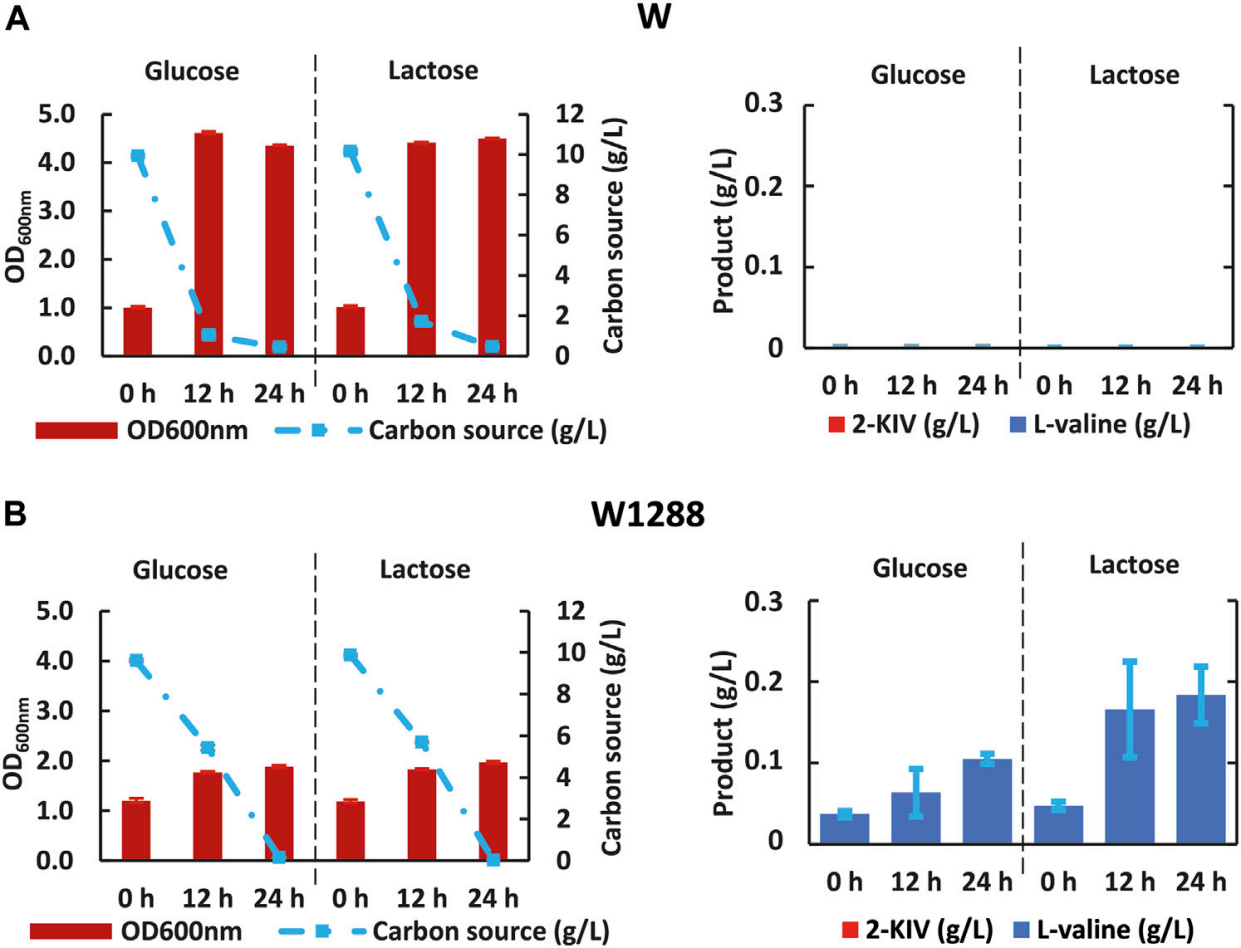
Kinetic of growth, substrate uptake and 2-ketoisovalerate and L-valine production of *E. coli* W **(A)** and *E. coli* W1288 **(B)** in shake flask experiments using glucose and lactose. Error bars indicate the difference between replicate cultures.

First we rerouted carbon flux towards pyruvate using scarless genome editing (Kim et al., 2014) to sequentially delete *ace*F and *mdh*. We then monitored growth performance of the resulting *E. coli* W1288 strain using glucose and lactose as sole carbon sources and compared it with the parental strain. We found significant differences in the engineered strain’s growth performance irrespective of the carbon source used in shake flask assays (Figure 6). For instance, *E. coli* W1288 exhibited lower substrate consumption and biomass production using glucose and lactose. As predicted *in silico,* deletion of *ace*F and *mdh* putatively resulted in lower levels of Acetyl-CoA in this phenotype and, therefore, TCA blockage thus explaining the growth performance observed. We registered significantly higher amounts of L-valine in cultures of *E. coli* W1288 using lactose as carbon source (up to 0.2 g/L) (Figure 6). This behavior was in line with *in silico* predictions and it strongly suggested that the double deletion increased flux through the BCAA pathway.

### 3.4 Engineering a 2-KIV *E. coli* W overproducer strain

**FIGURE 7:**
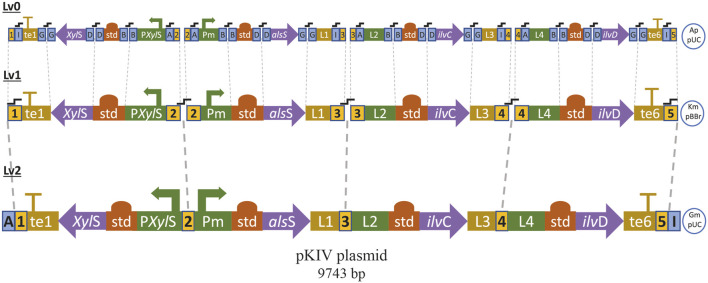
Sequential modular assembly of pKIV plasmid from basic DNA parts by using Golden Standard (See method). te1: T1 Terminator, L1, L2, L3 and L4: linker terminals and promoters, te6: BBa B1006 Terminator, std: consensus RBS, Ap: ampicillin resistance, Km: kanamycin resistance, Gm: gentamycin resistance, 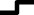
fusion sites of BsaI (blue square) and BpiI (yellow square) enzymes.

We assembled a synthetic pathway to express genes *als*S, *ilv*C and *ilv*D under heterologous expression driven by the XylS/*Pm* system (Figure 7). *ilv*C and *ilv*D encode the ketol-acid reductoisomerase and dihydroxy-acid dehydratase, which are responsible for synthetizing 2-KIV from acetolactate, while *als*S from *Bacillus subtilis* encodes a L-valine-insensitive acetolactate synthase broadly used for L-valine production (Felpeto-Santero et al., 2015; Hao et al., 2020). The synthetic pathway was constructed using Golden Standard technology (Blázquez et al., 2022) in conjunction with a high-copy-number host vector (Figure 7). The resulting plasmid, pKIV, was further expressed in wild-type *E. coli* W and *E. coli* W1288, thus yielding the *E. coli* W1294 and *E. coli* W1262 strains, respectively (Figure 5).

**FIGURE 8:**
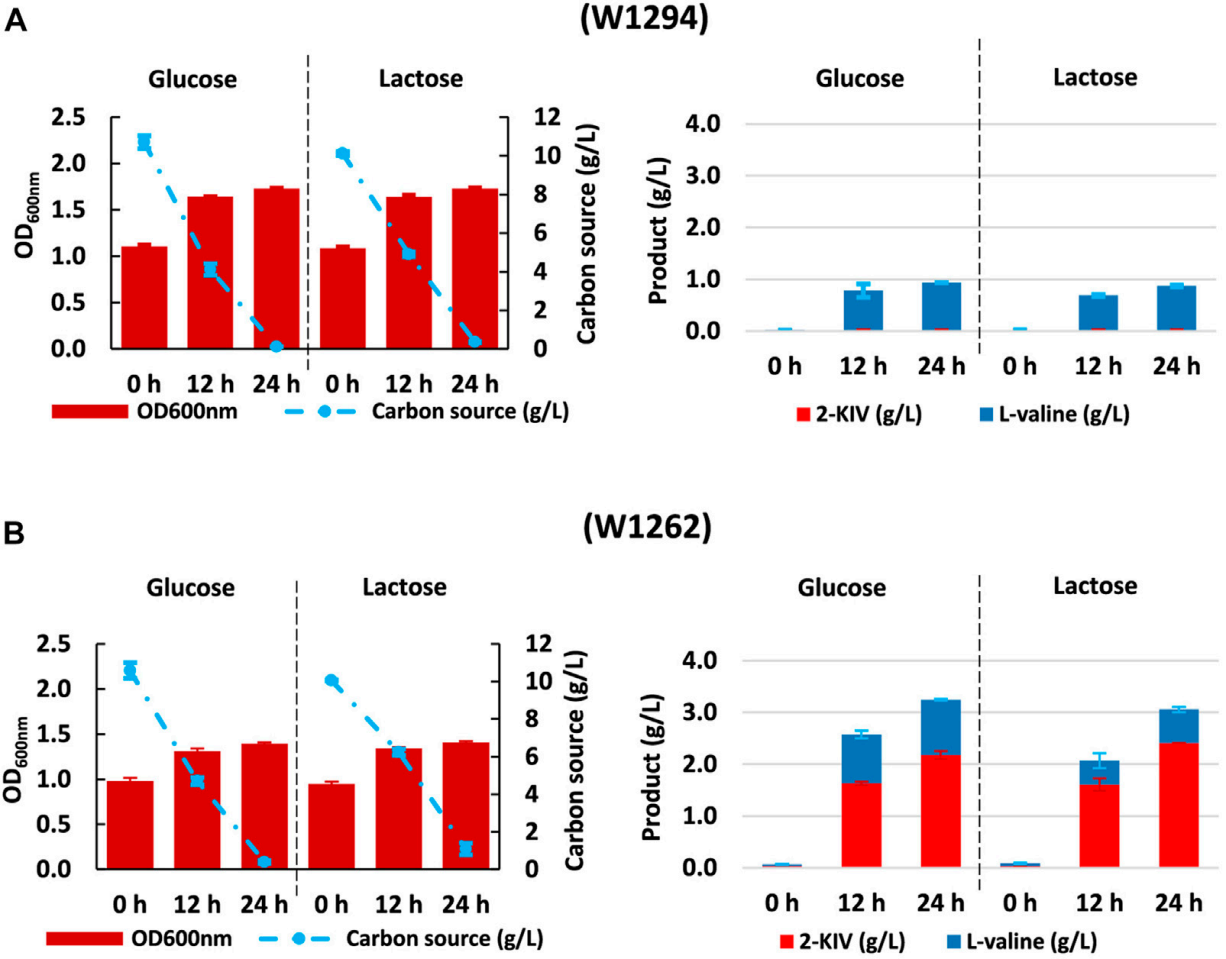
Kinetic of Growth, substrate uptake and 2-ketoisovalerate and L-valine production of *E. coli* W1294 **(A)** and *E. coli* W1292 **(B)** in shake flask experiments using glucose and lactose. Error bars indicate the difference between replicate cultures.

Despite no toxicity due 2-KIV has been previously reported (Zhou et al., 2022), we found that irrespective of the carbon source, expression of pKIV placed a metabolic burden on carrier strains that appeared to be greater in the wild-type genetic background of *E. coli* W1294 (Figure 8). Under production conditions, final biomass of W1294 was halved, while only a slight reduction was observed with the W1262 strain (Figures 6, 8). Beyond the potential metabolic burden induced by the replication and maintenance of pKIV (Fakruddin et al., 2013), we noticed a significant reduction in W1294s substrate consumption compared to the wild-type strain which contributed to its poor growth performance. Production of L-valine in large amounts (around 1 g/L) is likely the reason behind W1294s poorer growth performance and it suggests the absence of feedback inhibition of AHAS by L-valine using *als*S from *B. subtilis.*

Regarding the W1262 strain, we observed significant levels of L-valine production with minimal changes in growth and substrate consumption (Figure 8). Interestingly, the combination of carbon rerouting towards pyruvate, overexpression of the 2-KIV pathway and removal of feedback inhibition concurring in strain W1262 resulted in large titers of 2-KIV (2.2 ± 0.08 with glucose and 2.4 ± 0.00 g/L with lactose) (Figure 8C). In addition, we did not found side compounds such as acetate, formate and alcohols in the supernatants. The absence of byproducts suggests that 1) all the pyruvate available was funneled to 2-KIV and L-valine and 2) genetic modifications triggered no evident metabolic overflows, which is in agreement with *in silico* predictions.

### 3.5 Production of 2-KIV from non-conventional feedstock using non-conventional microbial cell factories

**FIGURE 9:**
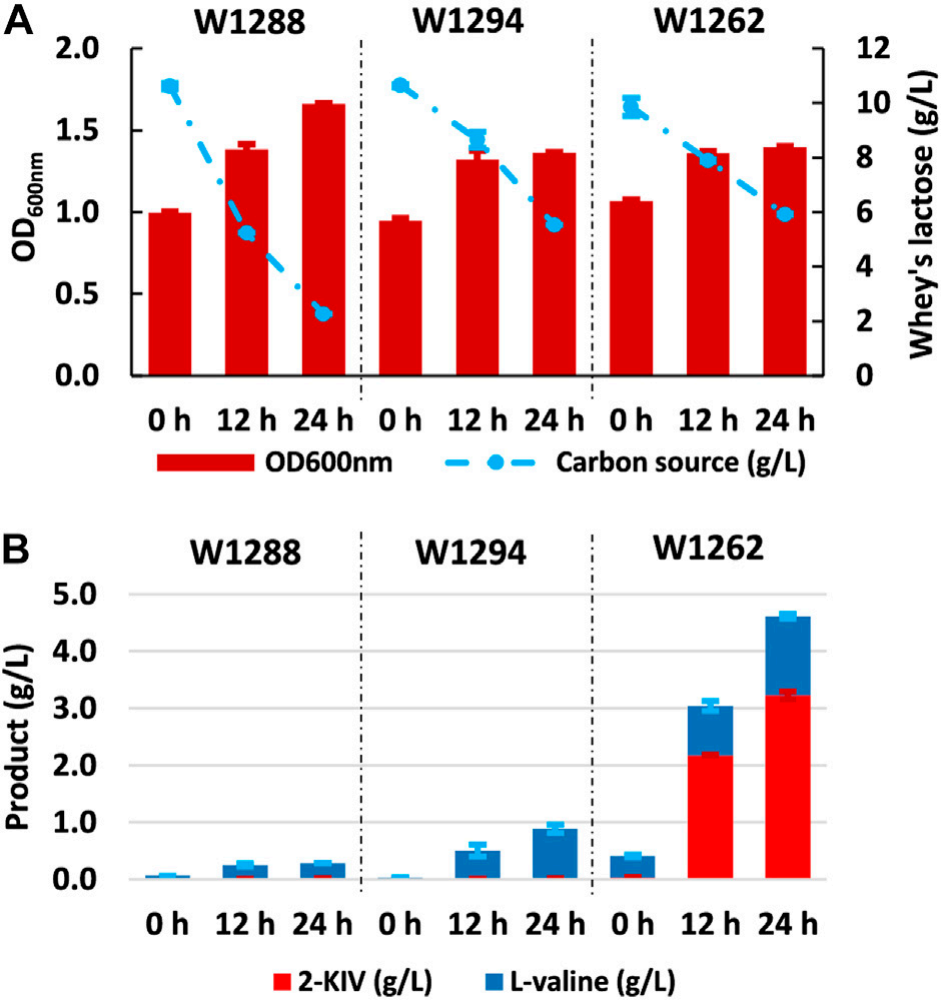
Kinetic of growth, substrate uptake and production of 2-ketoisovalerate and L-valine with *E. coli* W1288, *E. coli* W1294 and *E. coli* W1262 in shake flask experiments using whey’s lactose. Biomass production and carbon source uptake **(A)**, biosynthesis of products **(B)**. Error bars indicate the difference between replicate cultures.

The main aim of our work was to produce 2-KIV efficiently using whey (specifically the lactose contained in whey - see Materials and methods) as a non-conventional feedstock. To this end, we assessed the growth performance and 2-KIV production potential of strains W1288, W1294 and W1262 using whey lactose as sole carbon and energy source. Overall, we observed minor changes in terms of growth and L-valine production compared with the previous experiments performed with glucose and lactose. It was also noteworthy that the lactose in the whey was not fully consumed, especially not by strains harboring the pKIV plasmid, i.e., W1294 and W1262 (Figure 9). In what seems to be the corroboration of the suitability of whey lactose as an optimal carbon source for *E. coli* W, we found that strain W1262 delivered the greatest titers (3.22 ± 0.07 g/L 2-KIV and 1.40 ± 0.04 g/L L-valine) with whey lactose as carbon source, it is worth mentioning that, in all cases, a pH reduction in the medium between 6-6.5 was observed, which suggests that using a buffered medium with greater ionic strength would help to maintain a stable pH. Overall, we obtained up to 4.62 g/L of products with 3.9 g/L of whey lactose (Figure 8). These high yields are probably related to the presence of additional nutrients in the whey, i.e., up to 0.06% protein and 0.02% fat (Carranza-Saavedra et al., 2021; Tsermoula et al., 2021). Altogether, these results highlight the potential of whey lactose as feedstock for value-added compounds derived from pyruvate, such as 2-KIV and L-valine.

Finally, we compared our results with previously published works where authors had used alternative microbial cell factories and feedstocks (Table 2). Although a direct comparison is challenging due to differing methodologies and operation modes, we found that product yields per unit of biomass using *E. coli* W1262 exceeds yields reported for *K. pneumoniae*, *C. glutamicum* and *E. coli* B0016 irrespective of the carbon source, i.e., glucose, lactose and whey lactose (Table 2). Despite recording lower substrate-to-product conversion yields (Yp/s) with glucose and pure lactose than in recent studies with *P. putida* and *E. coli* B0016 (Batianis et al., 2022; Zhou et al., 2022), in the presence of whey lactose we achieved the highest Yp/s reported so far. This strongly highlights the potential of whey, a non-conventional feedstock, as a promising carbon source with the necessary potential to reduce production costs in microbial fermentation processes.

**TABLE 2:** Production of 2-KIV by different strains.

Strain	Operation mode	Medium	Time (h)	Total substrate uptake	2-KIV (g/L)	Yx/s (g/g)	Yp/s (g/g)	Yp/x (g/g)	References
*Corynebacterium glutamicum*	Fed-batch bioreactor	CGXII	56	82 g/L glucose approx.; 24 g/L potassium acetate approx	25.56	0.20	0.31	1.58	Krause et al. (2010)
		20 g/L ammonium sulfate; 1 g/L yeast extract; 5 g/L urea; 0.2 mg/L biotin							
*Corynebacterium glutamicum*	Fed-batch bioreactor	CGXII	44	82 g/L glucose approx.; 5 g/L potassium acetate approx	35.00	0.14	0.18	1.34	Buchholz et al. (2013)
		10 g/L yeast extract; 10 mM L-valine, L-isoleucine and L-leucine							
*Klebsiella pneumoniae*	Batch bioreactor	5 g/L yeast Extract; 4 g/L corn steep liquor; 5 g/L (NH4)2SO4; 0.4 g/L KCl and 0.1 g/L MgSO_4_	26	81 g/L glucose approx	17.40	0.05	0.21	4.68	Gu et al. (2017)
*Pseudomona putida*	Batch shake flask	M9	24	12 g/L glucose approx.; 0.12 g/L acetate approx	0.81	0.07 approx	0.40	0.95 approx	Batianis et al. (2022)
*E. coli* B0016	Fed-batch bioreactor	M9	26	130 g/L glucose approx	55.8	0.15 approx	0.55	2.93 approx	Zhou et al. (2022)
		5 g/L yeast extract; fed with 90 g/L yeast extract and 15 g/L peptone							
*E. coli* W1262	Batch shake flask	M9	24	10.2 g/L glucose	2.18	0.04	0.21	5.17	This study
		1 g/L yeast extract							
*E. coli* W1262	Batch shake flask	M9	24	9.0 g/L lactose	2.41	0.05	0.27	5.15	This study
		1 g/L yeast extract							
*E. coli* W1262	Batch shake flask	M9	24	3.9 g/L whey’s lactose	3.22	0.08	0.81	9.71	This study
		1 g/L yeast extract							

## 4 Conclusion

In this study we implemented an iterative approach to the production of valuable building blocks such as 2-KIV using non-conventional feedstocks and cell factories, i.e., whey lactose and the non-model *E. coli* W strain, and we demonstrated the latter’s suitability to deal with complex mixes of mono and disaccharides. In order to reduce the operational costs of the bioprocess, we designed a microbial chassis free of autotrophies and rich media requirements for growth and we rerouted the carbon flux towards pyruvate. Finally, we applied cutting-edge synthetic biology to the construction of a recombinant *E. coli* W strain capable of overproducing large titers of 2-KIV at the highest Yp/s reported so far. In what is a perfect fit for the demands of the circular economy, our approach and the W1262 strain pave the way for cost-effective production of key building blocks using recalcitrant feedstocks.

## Data Availability

The original contributions presented in the study are included in the article/Supplementary Material, further inquiries can be directed to the corresponding author.
