# Supplementary material for: System metabolic engineering of Escherichia coli W for the production of 2-ketoisovalerate using unconventional feedstock
Source: Front Bioeng Biotechnol. 2023 Apr 20;11:1176445. doi: 10.3389/fbioe.2023.1176445 (PMC10158823; doi:10.3389/fbioe.2023.1176445)
Supplement: Supplementary file 1 [file DataSheet1.docx]

Supplementary Table S1. Primers for making and verification of DNA cassette and DNA deletions

| Name | Sequences 5’ – 3’ | Source |
| --- | --- | --- |
| aceF_PCR_for | GCGAGATGGTCAGGATTGTGAACG | This study |
| aceF_PCR_rev | CCTAAATCAGCGCAACGGAAGG | This study |
| mdh_PCR_for | AGTCACCCGATATGGTGGTTG | This study |
| mdh_PCR_rev | ATGGATAAGCTGATCCGCGATG | This study |
| aceF_Rec_arm1_for | GACCGATTAATTAACTCCGGCAGTGGCATCTACCGACTATATG | This study |
| aceF_Rec_arm1_rev | GACCGAGGATCCCCGGCTCTTTTACTTACATCACCAGTGTCCGGTACTTTGATTTCGATAG | This study |
| acef_Rec_arm2_for | GACCGAGAGCTCGGCTATCGAAATCAAAGTACCGGACACTGGTGATGTAAGTAAAAGAGCCGG | This study |
| aceF_Rec_arm2_rev | GACCGAACTAGTCGCAGCGGCGTTAGCTTCAC | This study |
| mdh_Rec_arm1_for | GACCGATTAATTAAATTGAGAAACATGCCTGCGTCACGG | This study |
| mdh_Rec_arm1_rev | GACCGAGGATCCCTTATTAACGAACTCTTCGCCCAGGTTTTAACAGTAGTGCAAGCGCCTGG | This study |
| mdh_Rec_arm2_for | GACCGAGAGCTCCCAGGCGCTTGCACTACTGTTAAAACCTGGGCGAAGAGTTCGTTAATAAG | This study |
| mdh_Rec_arm2_rev | GACCGAACTAGTGGGATCGTGGTTAATGAAGTGTCAC | This study |

Supplementary Table S2. DNA used in this work

| DNA | Description - Sequences 5’ – 3’ | Source |
| --- | --- | --- |
| *Xyl*S activator, *Pm* promoter | GGAGTGCCCCTTTGGTCGAAAAAAAAAGCCCGCACTGTCAGGTGCGGGCTTTTTTCTGTGTTTCCACGTTCGTAATCAAGCCACTTCCTTTTTGCATTGACGCAGGGTGTCGGAAGGCAACTCGCCGAACGCGCTCCTATAGTTTTCAGCGAAGCGTCCCAAATGTAAGAAGCCGTAGTCTAGGGCTATCTCAGTTATACTACGCACATTGGCACTGGGATCGTTCAAGCAGGCGCGGATGCTTTCGAGCTTGCGGTTGCGGATGTAGTTCTTCGGCGTGGTGCCGGCGTGCTTCTCGAACAAATTGTAGAGCGAGCGTGGACTCATCATCGCCAGCTCCGCTAACCGCTCAAGGCTGATATTCCGTTTGAGATTCTCCTCAATGAATTGAACGACTCGCTCGAAAGACGGGTTACCTTTGCTGAAAATTTCACGGCTGACATTGCTGCCCAGCATTTCGAGCAGCTTGGAAGCGATGATCCCCGCATAGTGCTCTTGGACCCGAGGCATCGACTTTGTATGTTCCGCTTCGTCACAAACTAACCCGAGTAGATTGATAAAGCCATCGAGTTGCTGGAGATTGTGTCGCGCGGCGAAACGGATACCCTCCCTCGGCTTGTGCCAATTGTTGTCACTGCACGCCCGATCAAGGACCACTGAGGGCAATTTAACGATAAATTTCTCGCAATCTTCTGAATAGGTCAGGTCGGCTTGGTCATCCGGATTGAGCAGCAATAGTTCGCCCGGCGCAAAATAGTGCTCCTGGCCATGGCCACGCCACAGGCAATGGCCTTTGAGTATTATTTGCAGATGATAACAGGTTTCTAATCCAGGCGAGATTACCCTCACGCTACCGCCGTAGCTGATTCGACACAGATCGAGGCATCCGAAGATTCTGTGGTGCAGCCTGCCTGCCGGGCGCCCGCCCTTGGGCAGGCGAATAGAGTGCGTACCGACATACTGGTTAACATAATCGGAGACTGCATAGGGCTCGGCGTGGACGAAGATCTGACTTTTCTCGTTCAATAAGCAAAAATCCATAGTTCACGGTTCTCTTATTTTAATGTGGGCTGCTTGGTGTGATGTAGAAAGGCGCCAAGTCGATGAAAATGCATCTCGACGTGATGCGTATACGGGTTACCCCCATTGCCACGTTGCGCCATCCTTTTTGCAATCAGTGACCACTTTTCCAAGCAAAAATAACGCCAAGCAGAACGAACACGTTCTTTTTAAGAAGCGAGAACACCAGAAGTTCGTGCTGTCGGGGCATGGGGCGACGAATTGGCGGATAAAGGGGATCTGCTGGATATTACGGCCTTTTTAAAGACCGTAAAGAAAAATAAGCACAAGTTTTATCCGGCCTTTATTCACATTCTTGCCCGCCTGATGAATGCTCATCCGTAATTACGTATGGCAATGAAAGACGGTGAGCTGGTGATATGGGATAGTGTTCACCCTTGTTACACCGTTTTCCATGAGCAAACTGAAACGTTTTCATCGCTCTGGAGTGAATACCACGACGATTTCCGGCAGTTTCTACACATATATTCGCAAGATGTGGCGTGTTACGGTGAAAACCTGGCCTATTTCCCTAAAGGGTTTATTGAGAATATGTTTTTCGTCTCAGCCAATCCCTGGGTGAGTTTCACCAGTTTTGATTTAAACGTGGCCAATATGGACAACTTCTTCGCCCCCGTTTTCACCATGCATGGGAATTAGCTTGATCTGACCAACGACCGGTAGCGGAGCTATCCAACGGCGGTATACCAGGAAAACACACAGCAGGTACATCAGAACAGTACCATGACTGAAGAACAAATAGTTTTTTCCTGATCCATAAAGCAGAACGGCCTGCTCCATGACAAATCTGGCTCCCCAACTAATGCCCCATGCAGCCAGCATAACCAGCATAAAGGCAAGGAGTGCAGTGTCCGGTTTGATAGGGATAAGTCCAGCCTTGCAAGAAGCGGATACAGGAGTGCAAAAAATGGCTATCTCTAGTAAGGCCTACCCCTTAGGCTTTATGCAA | This study |
| *als*S | Gen extracted for PCR from *E. coli* W-pIZIbPSO | (Felpeto-Santero et al., 2015) |
| *ilv*D | Gen extracted for PCR from *E. coli* W-pIZIbPSO | (Felpeto-Santero et al., 2015) |
| *ilv*C | Gen extracted for PCR from *E. coli* W-pIZIbPSO | (Felpeto-Santero et al., 2015) |
| RBS-std | 1 | (Nogales et al., 2011) |
| T1 Terminator | GCTTCTTGGACTCCTGTTGATAGATCCAGTAATGACCTCAGAACTCCATCTGGATTTGTTCAGAACGCTCGGTTGCCGCCGGGCGTTTTTTATTGGTGAGAATCCAGCGCT | This study |
| BBa B1006 Terminator | AAAAAAAAACCCCGCCCCTGACAGGGCGGGGTTTTTTTT | http://parts.igem.org/Part:BBa_B1006 |
| Linker Terminator 1 | TCCCAGACCCACCTT | This study |
| Linker Terminator 3 | CACGGGCGGTAGCAG | This study |
| Linker  Promoter 2 | AAACTCAGTTGTAGT | This study |
| Linker  Promoter 4 | GGAGCCCCTGGCGCCCCTT | This study |
| Host plasmids | | |
| Lv1 Host vector | pSEVA23g19[g1] - Fusion sites 1AI2 | (Blázquez et al., 2022) |
|  | pSEVA23g19[g2] - Fusion sites 2AI3 |  |
|  | pSEVA23g19[g3] - Fusion sites 3AI4 |  |
|  | pSEVA23g19[g4] - Fusion sites 4AI5 |  |

Supplementary Table S3. designed DNA cassettes

| Name | Sequences 5’ – 3’ | Source |
| --- | --- | --- |
| Rec_arm1_aceF | CTCCGGCAGTGGCATCTACCGACTATATGAAACTGTTCGCTGAGCAGGTCCGTACTTACGTACCGGCTGACGACTACCGCGTACTGGGTACTGATGGCTTCGGTCGTTCCGACAGCCGTGAGAACCTGCGTCACCACTTCGAAGTTGATGCTTCCTACGTGGTTGTAGCGGCGCTGGGCGAACTGGCTAAACGTGGCGAAATCGATAAGAAAGTGGTTGCTGACGCAATCGCCAAATTCAACATCGATGCAGATAAAGTTAACCCGCGTCTGGCGTAAGAGGTAAAAGAATAATGGCTATCGAAATCAAAGTACCGGACACTGGTGATGTAAGTAAAAGAGCCGG | This study |
| Rec_arm2_aceF | GGCTATCGAAATCAAAGTACCGGACACTGGTGATGTAAGTAAAAGAGCCGGCCCAACGGCCGGCTTTTTTCTGGTAATCTCATGAATGTATTGAGGTTATTAGCGAATAGACAAATCGGTTGCCGTTTGTTGTTTAAAAATTGTTAACAATTTTGTAAAATACCGACGGATAGAACGACCCGGTGGTGGTTAGGGTATTACTTCACATACCCTATGGATTTCTGGGTGCAGCAAGGTAGCAAGCGCCAGAATCCCCAGGAGCTTACATAAGTAAGTGACTGGGGTGAGGGCGTGAAGCTAACGCCGCTGCG | This study |
| Rec_arm1_mdh | ATTGAGAAACATGCCTGCGTCACGGCATGCAAATTCTGCTTAAAAGTAAATTAATTGTTATCAAATTGATGTTGTTTTGGCTGAACGGTAGGGTATATTGTCACCACCTGTTGGAATGTTGCGCTAATGCATAAGCGACTGTTAATTACGTAAGTTAGGTTCCTGATTACGGCAATTAAATGCATAAACGCTAAACTTGCGTGACTACACATTCTTGAGATGTGGTCATTGTAAACGGCAATTTTGTGGATTAAGGTCGCGGCAGCGGAGCAACATATCTTAGTTTATCAATATAATAAGGAGTTTAGGATGAAAGTCGCAGTCCTCGGCGCTGCTGGCGGTATTGGCCAGGCGCTTGCACTACTGTTAAAACCTGGGCGAAGAGTTCGTTAATAAG | This study |
| Rec_arm2_mdh | CCAGGCGCTTGCACTACTGTTAAAACCTGGGCGAAGAGTTCGTTAATAAGTAATTAATTAGCGAATAATAAAAAACCGGAGCACAGACTCCGGTTTTTTGTTTTGAGCACTCGACTTAATTGGTTGCCGGATATTCCTGAATGGTGACCTGCAGCGTTAACTGCTTATCATCACGCATCACTACAACCGGGATCACCGAACCAGGGCGAATTTCTGCCACCTGATCCATCGTCTCCAGAGCAGAGATGGCCGGTTTGTTATCCACCGAAATAATCAGATCGTTGACCTGAATACCCGCATTCGCCGCCGGGCCGTCAGGTGACACTTCATTAACCACGATCCC | This study |
| Rec_kanamycin | GGATCCGCCCTGCTTAGAAAAACTCATCGAGCATCAAATGAAACTGCAATTTATTCATATCAGGATTATCAATACCATATTTTTGAAAAAGCCGTTTCTGTAATGAAGGAGAAAACTCACCGAGGCAGTTCCATAGGATGGCAAGATCCTGGTATCGGTCTGCGATTCCGACTCGTCCAACATCAATACAACCTATTAATTTCCCCTCGTCAAAAATAAGGTTATCAAGTGAGAAATCACCATGAGTGACGACTGAATCCGGTGAGAATGGCAAAAGCTTATGCATTTCTTTCCAGACTTGTTCAACAGGCCAGCCATTACGCTCGTCATCAAAATCACTCGCATCAACCAAACCGTTATTCATTCGTGATTGCGCCTGAGCGAGACGAAATACGCGATCGCTGTTAAAAGGACAATTACAAACAGGAATCGAATGCAACCGGCGCAGGAACACTGCCAGCGCATCAACAATATTTTCACCTGAATCAGGATATTCTTCTAATACCTGGAATGCTGTTTTCCCGGGGATCGCAGTGGTGAGTAACCATGCATCATCAGGAGTACGGATAAAATGCTTGATGGTCGGAAGAGGCATAAATTCCGTCAGCCAGTTTAGTCTGACCATCTCATCTGTAACATCATTGGCAACGCTACCTTTGCCATGTTTCAGAAACAACTCTGGCGCATCGGGCTTCCCATACAATCGATAGATTGTCGCACCTGATTGCCCGACATTATCGCGAGCCCATTTATACCCATATAAATCAGCATCCATGTTGGAATTTAATCGCGGCCTCGAGCAAGACGTTTCCCGTTGAATATGGCTCATAACACCCCTTGTATTACTGTTTATGTAAGCAGACAGTTTTATTGTTCATGATGATATATTTTTATCTTGTGCAATGTAACATCAGAGATTTTGAGACACAACGTGGCTTTGTTGAATAAATCGAACTTTTGCTGAGTTGAAGGATCAGATTACCCTGTTATCCCTAGCTGACACTGAGGATCTACTTACATACTGCTCGCGATAACTGACTCTGCCACTCAAGACCAAAAAAAACCCCGCCCTGTCAGGGGCGGGGTTTTTTTTTTGTTAGGTCAGTGACCTCTTAAGAGCGAAAAAACCCCGCCGAAGCGGGGTTTTTTGCGGGTAAGCGTCACTAACGACATTGGGTTCAGTTACTTAACAGAACCGCTGCCACTCTTGAGATAAGGGAGCTC | This study |

Supplementary Table S4. Reactions evaluated for Monte Carlo Sampling in *E. coli* W.

| **Gen** | **Enzyme** | **Reaction abbreviation** | **Reaction** |
| --- | --- | --- | --- |
| *pyk*AF | Pyruvate kinase | PYK | adp + H + pep -> pyr + atp |
| *ilv*BN, *ilv*GM*, ilv*IH | Acetolactate synthase | ACLS | H + 2pyr -> alac + CO_2_ |
| *ilv*D | Dihydroxy-acid dehydratase | DHAD1 | 23dhmb -> 2-KIV + H_2_O |
| *pho*E*, omp*FNC | Transport proteins | EX-2KIV | EX_2-KIV[Extra_organism] -> |
| *pho*E*, omp*FNC | Transport proteins | EX-valine | val_L[Extra_organism] -> |
| *mae*B | Malate dehydrogenase ubiquinone-8 | ME2 | mal_L + nadp -> nadph + CO_2_ + pyr |
| *ace*F | Pyruvate dehydrogenase | PDH | coa + nad + pyr -> CO_2_ + nadh + accoa |
| *ppc* | Phosphoenolpyruvate carboxylase | PPC | H_2_O + pep + CO_2_ -> pi + h + oaa |
| *mdh* | Malate dehydrogenase | MDH | mal_L + nad <=> h + nadh + oaa |
| *pho*E*, omp*FNC | Transport proteins | EX-acetate | ac [Extra_organism] -> |
| *tdc*E*, pfl*ABDC*, yfi*D | Pyruvate formate lyase | PFL | coa + pyr <=> accoa + for |
| *ace*A | Isocitrate lyase | ICL | icit -> glx + succ |
| *icd* | Isocitrate dehydrogenase - NADP | ICDHyr | icit + nadp <=> nadph + akg + CO_2_ |
| *suc*AB, *lpd* | Oxogluterato deshidrogenasa | AKGDH | akg + coa + nad -> CO_2_ + nadh + succoa |
| *acn*AB | Aconitase | ACONTb | acon + H_2_O <=> icit |
| *glt*A | Citrate synthase | CS | accoa + H_2_O + oaa -> cit + coa + H |

Supplementary Table S5. Maximum specific growth rates of *E. coli* W on different substrates pure and combined with glucose at a final concentration of 2 g/L.

| **Substrate (2 g/L)** | **µ_max_ (h^-1^)** |
| --- | --- |
| **Glucose 50% : Lactose 50%** | **0.824±0.01^a^**^b^ |
| Lactose | 0.82±0.11^ab^ |
| Glucose 50% : Fructose 50% | 0.81±0.02^abc^ |
| Glucose 50% : Maltose 50% | 0.73±0.01^abcd^ |
| Sucrose | 0.71±0.02^abcd^ |
| Glucose 50% : Sucrose 50% | 0.70±0.01^abcd^ |
| Maltose | 0.69±0.01^abcd^ |
| Galactose | 0.66±0.18^bcd^ |
| **Glucose** | **0.63±0.03^bcd^** |
| Fructose | 0.63±0.01^bcd^ |
| Glucose 50% : Galactose50% | 0.62±0.02^cd^ |

± represents the standard deviation. Similar lowercase letters per column indicate no statistical difference between treatments (p<0.05).

Supplementary Table S6. Studies on redirection the carbon flux towards pyruvate for the overproduction of 2-KIV, L-valine and some alcohols.

| **Microorganism** | **Genes Deletion** | **Product** | **Author** |
| --- | --- | --- | --- |
| *C. glutamicum* | *ilv*A, *pan*BC | L-valine | (Radmacher et al., 2002) |
| *E. coli* W3110 | *ilv*A, *leu*A, *pan*B, *ace*F, *md*h y *pfk*A | L-valine | (Park et al., 2007) |
| *C. glutamicum* | *ace*E | L-valine | (Blombach et al., 2007) |
| *E. coli* BW25113 | *adh*E, *ldh*A, *frd*BC – *fnr*, *pta* | Alcohols | (Atsumi et al., 2008) |
| *C. glutamicum* | *ace*E, *ace*E-*pqo, ace*E-*pqo-pgi* | L-valine | (Bartek et al., 2011) |
| *C. glutamicum* | *pan*B | L-valine | Holatko, 2009 |
| *C. glutamicum* | *ace*E, *pq*o, *ilv*E | 2-ketoisovalerate | (Krause et al., 2010) |
| *E. coli* W3110 | *ilv*A, *leu*A, *pan*B, *ace*F, *mdh* y *pfk*A | L-valine | (Park et al., 2011b) |
| *E. coli* W | *ilv*A, *lac*I | L-valine | (Park et al., 2011a) |
| *C. glutamicum* | *ace*E, *pqo*, *ilvE*, *ldh*A | Isobutanol | (Blombach et al., 2011) |
| *Bacillus subtilis* | *ldh*A | 2-ketoisovalerate e isobutanol | (Li et al., 2011) |
| *Saccharomyces cerevisiae* | *yqh*D | 2-ketoisovalerate decarboxylase | (Lee et al., 2012) |
| *Brevibacterium flavum* | *avt*A | L-valine | (Hou et al., 2012) |
| *C. glutamicum* | L-valina: *ace*E, *pqo*; *pqo*, *ppc*. 2-KIV: *ace*E, *pqo*, *ilv*E; *pqo*, *ppc*, *ilv*E | L-valine y 2-ketoisovalerate | (Buchholz et al., 2013) |
| *C. glutamicum* | *pep*C, *ldh*, *avt*A, *ctf*, *ack*A, *pta* | L-valine | (Hasegawa et al., 2013) |
| *C. glutamicum* | *ace*E, *ala*T, *ilv*A | L-valine | (Chen et al., 2015) |
| *Klebsiella pneumoniae* | *bud*A, *ldh*A, *bud*A-*ldh*A, *bud*A-*ldh*A-*Bud*B | 2-ketoisovalerate e isobutanol | (Gu et al., 2017) |
| *C. glutamicum* | *ppc*, *pyc* | L-valine | (Schwentner et al., 2018) |
| *E. coli* MG1655 | *pfl*B, *ldh*A, *ack*A-*pt*a | Isobutanol | (Liang et al., 2018) |
| *Bacillus subtilis* | *Bcd, ilvB, leuA, ilvA, pdhA, sigF* | L-valine | (Westbrook et al., 2018) |
| *E. coli* MG1655 | *pgi*, *gnt*R, *gn*d, *pfl*B, *ldh*A | Isobutanol | (Noda et al., 2019) |
| *E. coli* MG1655 | *frd*A, *pta*, *ldh*A, *adh*E | 2,3-butanediol and isobutanol | (Jung et al., 2020) |
| *E. coli* W3110 | *pfl*B, *adh*E y *ldh*A | L-valine | (Hao et al., 2020) |

Supplementary method descriptions MD1. Construction of knockout mutants

The deletion of *mdh* and *ace*F genes in *Escherichia coli* W was performed using the method developed by Kim *et al.* (Kim et al., 2014) with minor modifications: i) The T2SK from the plasmid pT2SK was adapted by PCR introducing *Sac*I and *Bam*HI restriction sites at both sides (T2SK cassette). Ii) 200 pb upstream and downstream of *mdh* and *ace*F (Rec_arms 1 and 2) were amplified by PCR using *E. coli* W chromosome as template and ligated at both sides of T2SK in the *Sac*I and *Bam*HI sites and cloned in pSEVA182 generating the mdh_Arm1y2 and aceF_Arm1y2 constructs (Figure S1 and Figure S2). iii) These constructs where used as PCR template for the construction of the final mutational cassettes for the generation of the knockout mutants.

Supplementary method descriptions MD2. Construction of synthetic expression systems

For the overexpression of desired genes synthetic operons were constructed according to the Golden Standard method developed in our lab (Blázquez et al., 2022). The CDS level 0 parts were constructed by chemical synthesis (Supplementary Table S2). Were used linkers for a continue transcription of genes. In addition, through of previous test of others ribosomes, was selected standard ribosome (std) (Nogales et al., 2011), everything this within a cloning vector of high copy number (pUC) and using 3MB at 0.5 mM to activate the expression system.

Supplementary Figure S1. Designed plasmid to deletion of *mdh* gen in *E. coli* W

Supplementary Figure S2. Designed plasmid to deletion of *ace*F gen in *E. coli* W


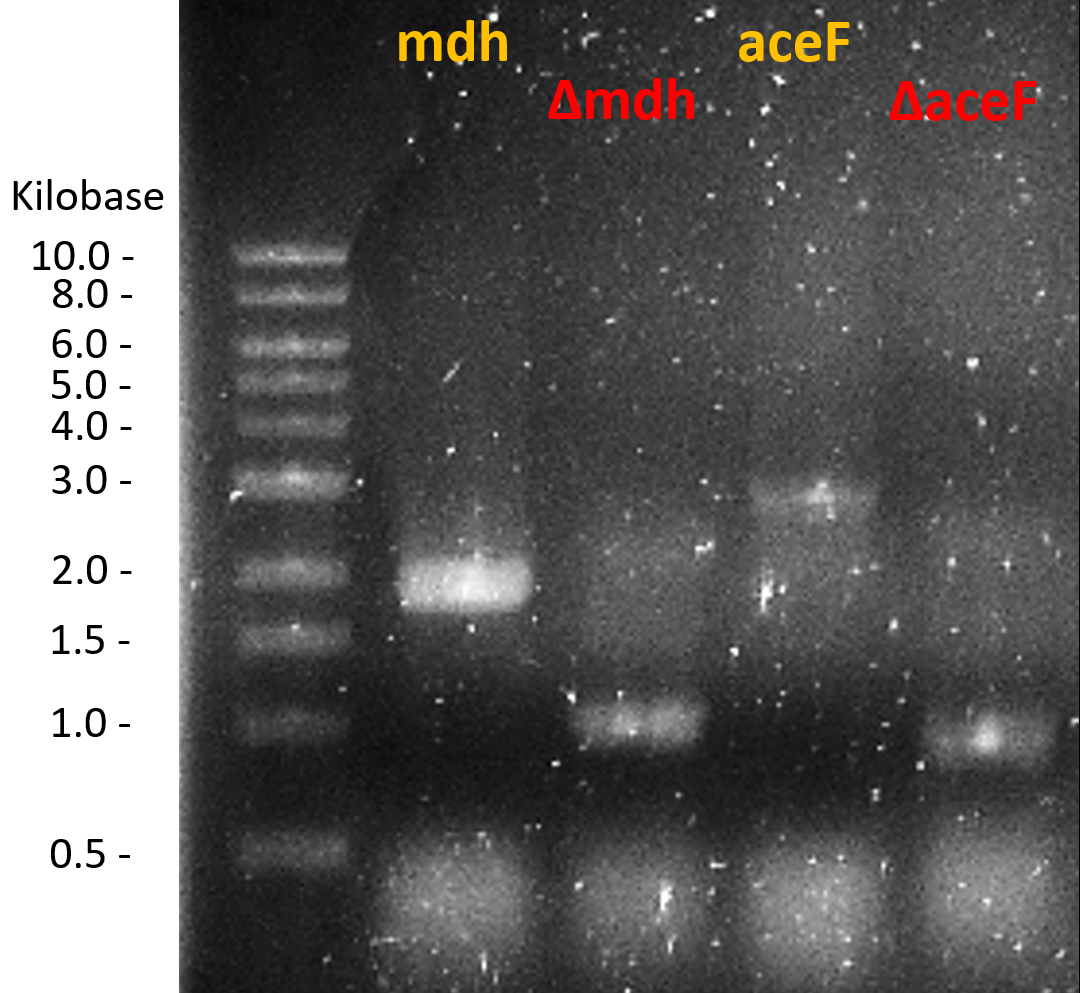


Supplementary Figure S3. Gel electrophoresis for verification of gene knockout in *E. coli* W


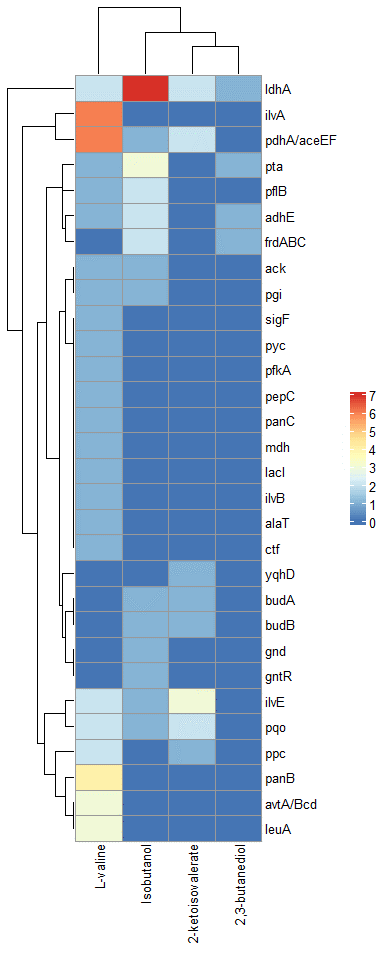


Supplementary Figure S4. Cluster of genes found to increase production of pyruvate, 2-KIV and L-valine

**References**

Atsumi, S., Hanai, T., Liao, J.C., 2008. Non-fermentative pathways for synthesis of branched-chain higher alcohols as biofuels. Nature 451, 86–89. https://doi.org/10.1038/nature06450

Bartek, T., Blombach, B., Lang, S., Eikmanns, B.J., Wiechert, W., Oldiges, M., Nöh, K., Noack, S., 2011. Comparative 13C metabolic flux analysis of pyruvate dehydrogenase complex-deficient, L-valine-producing *Corynebacterium glutamicum*. Appl. Environ. Microbiol. 77, 6644–6652. https://doi.org/10.1128/AEM.00575-11

Blázquez, B., Torres-Bacete, J., Leon, D.S., Kniewel, R., Martinez, I., Sordon, S., Wilczak, A., Salgado, S., Huszcza, E., Popłoński, J., Prieto, M.A., Nogales, J., 2022. Golden Standard: A complete standard, portable, and interoperative MoClo tool for model and non-model bacterial hosts. bioRxiv 2022.09.20.508659. https://doi.org/10.1101/2022.09.20.508659

Blombach, B., Riester, T., Wieschalka, S., Ziert, C., Youn, J.-W., Wendisch, V.F., Eikmanns, B.J., 2011. *Corynebacterium glutamicum* Tailored for Efficient Isobutanol Production. Appl. Environ. Microbiol. 77, 3300–3310. https://doi.org/10.1128/AEM.02972-10

Blombach, B., Schreiner, M.E., Holátko, J., Bartek, T., Oldiges, M., Eikmanns, B.J., 2007. L-valine production with pyruvate dehydrogenase complex-deficient *Corynebacterium glutamicum*. Appl. Environ. Microbiol. 73, 2079–2084. https://doi.org/10.1128/AEM.02826-06

Buchholz, J., Schwentner, A., Brunnenkan, B., Gabris, C., Grimm, S., Gerstmeir, R., Takors, R., Eikmanns, B.J., Blombacha, B., 2013. Platform engineering of *Corynebacterium glutamicum* with reduced pyruvate dehydrogenase complex activity for improved production of l-lysine, l-valine, and 2-ketoisovalerate. Appl. Environ. Microbiol. 79, 5566–5575. https://doi.org/10.1128/AEM.01741-13

Chen, C., Li, Y., Hu, J., Dong, X., Wang, X., 2015. Metabolic engineering of *Corynebacterium glutamicum* ATCC13869 for l-valine production. Metab. Eng. 29, 66–75. https://doi.org/10.1016/j.ymben.2015.03.004

Felpeto-Santero, C., Rojas, A., Tortajada, M., Galán, B., Ramón, D., García, J.L., 2015. Engineering alternative isobutanol production platforms. AMB Express 5. https://doi.org/10.1186/s13568-015-0119-2

Gu, J., Zhou, J., Zhang, Z., Kim, C.H., Jiang, B., Shi, J., Hao, J., 2017. Isobutanol and 2-ketoisovalerate production by *Klebsiella pneumoniae* via a native pathway. Metab. Eng. 43, 71–84. https://doi.org/10.1016/j.ymben.2017.07.003

Hao, Y., Ma, Q., Liu, X., Fan, X., Men, J., Wu, H., Jiang, S., Tian, D., Xiong, B., Xie, X., 2020. High-yield production of L-valine in engineered *Escherichia coli* by a novel two-stage fermentation. Metab. Eng. 62, 198–206. https://doi.org/10.1016/j.ymben.2020.09.007

Hasegawa, S., Suda, M., Uematsu, K., Natsuma, Y., Hiraga, K., Jojima, T., Inui, M., Yukawa, H., 2013. Engineering of *Corynebacterium glutamicum* for high-yield l-valine production under oxygen deprivation conditions. Appl. Environ. Microbiol. 79, 1250–1257. https://doi.org/10.1128/AEM.02806-12

Hou, X., Chen, X., Zhang, Y., Qian, H., Zhang, W., 2012. l-Valine production with minimization of by-products’ synthesis in *Corynebacterium glutamicum* and *Brevibacterium flavum*. Amin. Acids 2012 436 43, 2301–2311. https://doi.org/10.1007/S00726-012-1308-9

Jung, H.M., Han, J.H., Oh, M.K., 2020. Improved production of 2,3-butanediol and isobutanol by engineering electron transport chain in *Escherichia coli*. Microb. Biotechnol. 14, 213–226. https://doi.org/10.1111/1751-7915.13669

Kim, Webb, A.M., Kershner, J.P., Blaskowski, S., Copley, S.D., 2014. A versatile and highly efficient method for scarless genome editing in Escherichia coli and Salmonella enterica. BMC Biotechnol. 14. https://doi.org/10.1186/1472-6750-14-84

Krause, F.S., Blombach, B., Eikmanns, B.J., 2010. Metabolic engineering of *Corynebacterium glutamicum* for 2-Ketoisovalerate production. Appl. Environ. Microbiol. 76, 8053–8061. https://doi.org/10.1128/AEM.01710-10

Lee, W.H., Seo, S.O., Bae, Y.H., Nan, H., Jin, Y.S., Seo, J.H., 2012. Isobutanol production in engineered *Saccharomyces cerevisiae* by overexpression of 2-ketoisovalerate decarboxylase and valine biosynthetic enzymes. Bioprocess Biosyst. Eng. 35, 1467–1475. https://doi.org/10.1007/s00449-012-0736-y

Li, S., Wen, J., Jia, X., 2011. Engineering *Bacillus subtilis* for isobutanol production by heterologous Ehrlich pathway construction and the biosynthetic 2-ketoisovalerate precursor pathway overexpression. Appl. Microbiol. Biotechnol. 91, 577–589. https://doi.org/10.1007/s00253-011-3280-9

Liang, S., Chen, H., Liu, J., Wen, J., 2018. Rational design of a synthetic Entner–Doudoroff pathway for enhancing glucose transformation to isobutanol in *Escherichia coli*. J. Ind. Microbiol. Biotechnol. 45, 187–199. https://doi.org/10.1007/s10295-018-2017-5

Noda, S., Mori, Y., Oyama, S., Kondo, A., Araki, M., Shirai, T., 2019. Reconstruction of metabolic pathway for isobutanol production in *Escherichia coli*. Microb. Cell Fact. 18, 124. https://doi.org/10.1186/s12934-019-1171-4

Nogales, J., Canales, A., Jiménez-Barbero, J., Serra, B., Pingarrón, J., García, J., Díaz, E., 2011. Unravelling the gallic acid degradation pathway in bacteria: the gal cluster from *Pseudomonas putida*. Mol. Microbiol. 79, 359–374. https://doi.org/10.1111/J.1365-2958.2010.07448.X

Park, J.H., Jang, Y., Lee, J.W., Lee, S.Y., 2011a. Escherichia coli W as a new platform strain for the enhanced production of L-Valine by systems metabolic engineering 108, 1140–1147. https://doi.org/10.1002/bit.23044

Park, J.H., Kim, T.Y., Lee, K.H., Lee, S.Y., 2011b. Fed-batch culture of *Escherichia coli* for L-valine production based on in silico flux response analysis. Biotechnol. Bioeng. 108, 934–946. https://doi.org/10.1002/bit.22995

Park, J.H., Lee, K.H., Kim, T.Y., Lee, S.Y., 2007. Metabolic engineering of *Escherichia coli* for the production of L -valine based on transcriptome analysis and in silico gene knockout simulation. PNAS 104, 7797–7802. https://doi.org/10.1073/pnas.0702609104

Radmacher, E., Vaitsikova, A., Burger, U., Krumbach, K., Sahm, H., Eggeling, L., 2002. Linking central metabolism with increased pathway flux: L-valine accumulation by *Corynebacterium glutamicum*. Appl. Environ. Microbiol. 68, 2246–2250. https://doi.org/10.1128/AEM.68.5.2246-2250.2002

Schwentner, A., Feith, A., Münch, E., Busche, T., Rückert, C., Kalinowski, J., Takors, R., Blombach, B., 2018. Metabolic engineering to guide evolution – Creating a novel mode for L-valine production with *Corynebacterium glutamicum*. Metab. Eng. 47, 31–41. https://doi.org/10.1016/j.ymben.2018.02.015

Westbrook, A.W., Ren, X., Moo-Young, M., Chou, C.P., 2018. Metabolic engineering of *Bacillus subtilis* for l-valine overproduction. Biotechnol. Bioeng. 115, 2778–2792. https://doi.org/10.1002/bit.26789
